# Neuropsychological Effects of Cannabis Use in Adolescent Populations: A Case Based Literature Review

**DOI:** 10.1192/j.eurpsy.2026.10821

**Published:** 2026-07-31

**Authors:** I. S. Ruisánchez, C. C. Massé, E. A. Torres, G. C. Vivó, C. L. Montealegre

**Affiliations:** Psychiatry, Hospital Clinic, Barcelona, Spain

## Abstract

**Introduction:**

Cannabis use among adolescents is a phenomenon that has increased in recent years, raising concern within the scientific and medical communities due to its potential neuropsychological effects. During adolescence, the brain is still developing, making it particularly vulnerable to psychoactive substances such as cannabis.

**Objectives:**

1. To analyze the possible neuropsychological consequences of early cannabis use, focusing on the adolescent population.
2. To study the brain regions involved in these behavioral and psychological alterations.

**Methods:**

A narrative literature review was conducted to synthesize current evidence regarding the neuropsychological effects of cannabis use in adolescent and young populations and to contextualize a related clinical case. Literature searches were performed in PubMed and Google Scholar using the keywords *“cannabis,” “adolescents,” “young,” “neuropsychology,” “neuroimaging,”* and *“effects.”* No publication date restrictions were applied. Studies were included if they examined neuropsychological, cognitive, neuroimaging, or other relevant effects associated with cannabis exposure in adolescents or young individuals. Studies focusing exclusively on adult populations or on non-neuropsychological variables were excluded.

A total of fifteen relevant publications meeting inclusion criteria were selected and critically appraised. Findings were integrated qualitatively to identify convergent evidence, methodological limitations, and implications for neuropsychological assessment and clinical interpretation in the context of adolescent cannabis use.

**Results:**

Cannabis use during adolescence is associated with deficits in social cognition, executive control, and attention problems. Additionally, it impacts inhibitory control, working memory, and processing speed compared to non-users. These deficits may persist even after abstinence. Neuroimaging evidence indicates that cannabis use alters activation in prefrontal and cingulate regions, disrupting networks involved in executive, inhibitory, and emotional control. Reduced activation in frontal and cingulate areas is associated with poorer inhibition, working memory, and emotion regulation, while increased posterior cingulate connectivity relates to subjective “high” sensations. These findings suggest functional reorganization of neural circuits underlying cognitive control in cannabis users.

**Conclusions:**

This review highlights that cannabis use during adolescence can significantly impact neuropsychological development, affecting cognitive functions such as memory, attention, and social cognition, among others. Early detection and preventive strategies are emphasized to mitigate long-term effects. It is crucial to continue research and raise awareness about the associated risks, promoting prevention and support programs for this vulnerable population.

**Disclosure of Interest:**

None Declared

